# Bupropion for the treatment of fluoxetine non-responsive trichotillomania: a case report

**DOI:** 10.1186/1752-1947-5-557

**Published:** 2011-11-30

**Authors:** Rajshekhar Bipeta, Srinivasa SRR Yerramilli

**Affiliations:** 1 Rajasri Clinic, Anandbagh Crossroads, Malkajgiri, Hyderabad, Andhra Pradesh, India; 2 Sri Venkateswara Nursing Home, Narayanaguda, Hyderabad, Andhra Pradesh, India

## Abstract

**Introduction:**

Trichotillomania, classified as an impulse control disorder in the *Diagnostic and Statistical Manual of Mental Disorders*, is characterized by the recurrent pulling out of one's hair, resulting in noticeable hair loss. The condition has a varied etiology. Specific serotonin reuptake inhibitors are considered the treatment of choice; however some patients fail to respond to this class of drugs. A few older reports suggest possible benefit from treatment with bupropion.

**Case presentation:**

A 23-year-old Asian woman with fluoxetine non- responsive trichotillomania was treated with sustained release bupropion (up to 450 mg/day) and cognitive behavior therapy. She demonstrated clinically significant improvement on the Clinical Global Impression - Improvement scale by week 13. The improvement persisted throughout the 12-month follow-up period.

**Conclusions:**

The present case report may be of interest to psychiatrists and dermatologists. Apart from the serotonergic pathway, others, such as the mesolimbic pathway, also appear to be involved in the causation of trichotillomania. Bupropion may be considered as an alternative pharmacological treatment for patients who do not respond to specific serotonin reuptake inhibitors. However, this initial finding needs to be confirmed by well designed double-blind placebo controlled trials.

## Introduction

Trichotillomania (TTM) is characterized by a compulsive urge to pull out one's own hair, leading to noticeable hair loss, distress, and social or functional impairment. TTM is usually confined to one or two sites, mainly the scalp, eyebrows, and eyelashes, but can also involve other parts of the body.

TTM is classified as an impulse control disorder in the *Diagnostic and Statistical Manual of Mental Disorders*, fourth edition (DSM-IV), and is believed to be related to obsessive-compulsive spectrum disorders [1,2]. Patients with obsessive-compulsive disorder (OCD) and patients with TTM share overlapping comorbidity, familial transmission, treatment response, and difficulties in suppressing inappropriate repetitive behaviors, indicating that there is an underlying dysregulation in inhibitory control processes [3].

OCD has a fluctuating course with exacerbations and remissions, and the symptoms change over time in terms of focus (for example, ritualistic washing, checking stoves, and so on). Unlike OCD, TTM has both impulsive as well as compulsive elements. It does not occur in response to obsessive thoughts, but rather because of an irresistible urge to pull hair at leisure and the sense of satisfaction when the hair is pulled. Additionally, patients with TTM usually present only with hair pulling without evolution to other compulsive rituals, and usually do not have a fluctuating course. Depression and anxiety is less in TTM compared with OCD. Long-term response to treatment with specific serotonin reuptake inhibitors (SSRIs) is consistent in OCD while it is variable in TTM [4-6].

Genetic, classificatory and pharmacological treatment studies have suggested the possibility of addictive behavior in TTM [4]. As in other addictive disorders (such as pathological gambling), bupropion may be considered in the management of TTM, as there is emerging evidence for the role of dopamine pathways in this condition [3].

## Case presentation

Our patient was a 23-year-old unmarried, unemployed woman graduate from a middle socioeconomic status and urban Asian background. She had a six-year history of patchy hair loss from her scalp. She had earlier consulted a dermatologist who referred her to a psychiatrist.

She walked into our consultation room with her head covered with a scarf. She had a short haircut and there were many bald patches, 5 cm to 10 cm in area, on her scalp. The hair, particularly in the parietal region of both sides of the head, was very thin, brittle and uneven.

Our patient had an uncontrollable, irresistible, repetitive urge to pull out her scalp hair. The hair-pulling behavior had first appeared when she was 17 years of age, had completed school and was entering college. There was no evidence of any significant stressors in her life. The hair pulling had become particularly distressing and problematic over the past six months and she described her present condition as 'tremendously out of control'. There was a significant deterioration in her academic performance and social functioning.

The hair pulling spells occurred on a daily basis, just before bedtime, when she was alone in her room listening to the radio, or watching television. The hair pulling used to be more severe when she was stressed. She reported a feeling of mounting tension before the act of hair pulling, with an accompanied sensation of itching. The tension was alleviated when she pulled the hair out. Pulling of hair involved twisting each hair around a finger, mostly from the sides of her head near her ears. She reported a sense of satisfaction in hearing the sound of the hair being pulled out, accompanied by pleasurable sensations. She would pull out countless hairs over few hours, resulting in a bald patch on the scalp. After the hair was pulled, she would examine the root of the hair. Later, she would secretly dispose of the plucked out hair after neatly packing them in a cover.

She felt guilty and embarrassed by her hair-pulling behavior and often wore her hair tied back. She was more self-conscious about the bald patches behind the ears. She used scarves to camouflage her disfigured scalp while going out. She had good insight into her illness, and recognized that her mental state was contributing to her symptoms.

Our patient was pre-morbidly well adjusted with no significant medical or family history. There was also no history of substance use or suicide attempts. She was diagnosed to have TTM (impulse control disorder not otherwise specified) as per DSM-IV criteria [1]. There was no comorbid Axis I psychiatric disorders, including mood disorder. OCD was ruled out in our patient as the hair pulling (i) used to occur in leisure time only, (ii) was not in response to obsessive thoughts, but rather because of irresistible urges, (iii) was followed by a sense of satisfaction, (iv) was not associated with any other OCD symptoms, and (v) was not associated with depression.

At various time points, different instruments were applied to measure her treatment response (Table 1). TTM behavior was measured with The Massachusetts General Hospital Hair Pulling Scale (MGHHPS) and National Institute of Mental Health Trichotillomania Symptom Severity and Impairment Scale (NIMH-TSS and NIMH-TIS) [5,6]. MGHHPS is a self-report measure of the severity of hair pulling urges and behavior, efforts to resist urges, control over the problem, and associated distress [5]. Higher scores reflect greater severity. The NIMH-TSS and NIMH-TIS are clinician rating scales [6]. The NIMH-TSS is a five-item measure assessing average hair-pulling episode duration during the past week and on the previous day, thoughts preceding the pulling episode, resistance to urges, distress, and interference. The NIMH-TIS is an 11-point scale assessing overall impairment in a patient's life due to TTM. Clinical Global Impression - Severity and Improvement scales (CGI-S and CGI- I) are clinician-rated instruments to assess the baseline severity and improvement in a psychiatric disorder [7]. Presence of depression was assessed with the Hospital Anxiety Depression Rating Scale - Depression subscale (HADS-D) [8].

**Table 1:** Assessments at various time points throughout the 12-month follow-up period

Scales/score range	Week 0	Week 8	Week 12	Week 13	Week 24 (6 months)	Week 48 (12 months)
CGI-S (1-7)	4, moderate symptoms	4, moderate symptoms	4, moderate symptoms	2, borderline mentally ill	2, borderline mentally ill	2, borderline mentally ill
CGI-I (1-7)	NA	4, no change	3, mild improvement	2, much improvement	2, much improvement	1, very much improved
MGHHPS (0-28)	21, moderate symptoms	20, moderate symptoms	18, moderate symptoms	10, mild symptoms	9, mild symptoms	9, mild symptoms
NIMH-TSS (0-30)	23, moderate symptoms	22, moderate symptoms	19, moderate symptoms	12, mild symptoms	10, mild symptoms	8, mild symptoms
NIMH-TIS (0-10)	8, moderate-severe impairment	8, moderate-severe impairment	7, moderate-severe impairment	3, minimal impairment	2, minimal impairment	1, minimal impairment
HADS-D (0-21)	5, normal: no depression	-	-	-	-	3, normal: no depression

CGI-S/I = Clinical Global Impression - Severity/Improvement; HADS-D = Hospital Anxiety Depression Rating Scale - Depression; MGHHPS = Massachusetts General Hospital Hair Pulling Scale; NIMH-TIS/TSS = National Institute of Mental Health Trichotillomania Impairment Scale/Trichotillomania Symptom Severity Scale.

**Figure 1:**
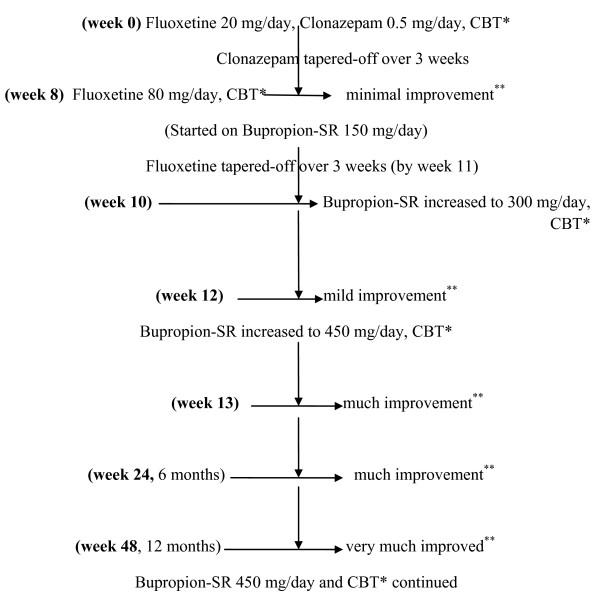
**Flowchart depicting the sequence of events in our patient's case**. *CBT = Cognitive Behavior Therapy **Improvement based on Clinical Global Impression - Improvement (CGI-I) scale scores

Our patient was initially started on fluoxetine 20 mg/day (gradually increasing the dose up to 80 mg/day by week eight), clonazepam 0.5 mg/day (tapered off by week three) and continuous cognitive behavior therapy (CBT). By week eight, despite the reasonably adequate dose and treatment duration, there was minimal improvement in hair-pulling behavior. Hence, at week eight, bupropion sustained release (bupropion-SR) was initiated at 150 mg/day, and increased to 300 mg/day at week 10. Fluoxetine was tapered off by week 11. By week 12, there was a mild improvement in our patient's hair pulling behavior, and the dose of bupropion-SR was increased to 450 mg/day, in divided doses. At this dose, at week 13, our patient experienced significant clinical improvement in TTM. She continued to show sustained improvement during the 6 and 12-month follow-ups (Figure 1). CBT was started at the beginning of treatment, and was continued throughout the one-year follow-up period. Bupropion-SR was well tolerated, except for few mild adverse effects such as nausea and nervousness during the initial two weeks, which resolved without any intervention.

## Discussion

Our patient was initially treated with fluoxetine, a specific serotonin reuptake inhibitor (SSRI), up to 80 mg/day for adequate duration along with CBT, but there was minimal improvement. Very few case reports are available in the published literature about the use of bupropion in the treatment of TTM. In our patient, bupropion-SR was given up to 450 mg/day, the dosage used in other studies [3,9,10]. Bupropion-SR was well tolerated and was beneficial in reducing the hair-pulling behavior. The observed improvement does not appear to be related to the anti-depressant effect of bupropion, since the HADS-D score was within normal limits at baseline and did not change significantly at the end of the one-year follow-up. Also, clinically, there was no evidence of depression throughout the follow-up period. Similar findings were reported by Dannon *et al*. [3].

TTM is proposed to be a heterogeneous disorder that overlaps with OCD, impulse control disorders and also addictive disorders. There is an association of TTM with attention deficit hyperactivity disorder and impulsive behavior [3].

Traditionally, serotonin has been the principal neurotransmitter implicated in TTM and other impulse control disorders. However, recent literature strongly supports the role of dopamine in the pathophysiology of TTM [10]. Lochner *et al*. have theorized that TTM is more impulsive than compulsive in nature [4]. TTM is not just an impulsive-compulsive disorder, but also appears to have an addictive component to it. Dannon *et al*. proposed that TTM (like addictive disorders) is characterized by compulsive seeking, and consequently obtaining the substance of choice (that is, hair), with progressive loss of behavior control [3]. Thus, like pathological gambling, TTM appears to be more like an addictive disorder, where the mesolimbic dopamine reward pathway and prefrontal cortex are implicated.

Thus, TTM may be conceptualized as an impulsive-compulsive-addictive spectrum disorder. It has been hypothesized that specific subtypes of TTM exist with specific patterns of treatment response [3,9]. Many pharmacological agents have been reported to be useful in TTM. These include SSRIs, anti-convulsants, anti-psychotics and naltrexone. Earlier studies, however, have failed to show efficacy of SSRI treatment [3].

Bupropion is useful in nicotine, caffeine, alcohol and other addictive disorders through its action on the mesolimbic pathways, where it inhibits the reuptake of dopamine and norepinephrine. There is now also evidence that bupropion similarly helps in TTM (and also pathological gambling) by diminishing the heightened arousal and pleasurable relief [10,11]. Hence, bupropion may be considered as an effective alternative in patients with TTM not responding to SSRIs.

There is definite evidence that CBT is beneficial in TTM, and the synergistic benefit of CBT in our patient cannot be undermined [12,13]. However, it should be noted that CBT was started from the beginning of the treatment along with fluoxetine, but significant improvement was observed only when the treatment was changed over to a combination of bupropion-SR and CBT. Thus, the therapeutic response appears more likely to be related to bupropion-SR.

## Conclusions

A single case report has associated limitations, especially when the clinician is not blinded to treatment allocation and assessments. Also, few of the scales (such as MGHHPS, NIMH-TSS and NIMH-TIS) are not standardized for the Indian population. However, these case reports do give insight into the nature of the disorders discussed, and also help explore new treatment options. The possibility that TTM may have an addictive component needs to be studied further. Bupropion may be considered as a treatment, especially in case of patients with TTM who are non-responders to SSRIs. Further well conducted randomized trials are required to evaluate the effectiveness of bupropion-SR in TTM and other impulsive-compulsive-addictive spectrum disorders.

## Consent

Written informed consent was obtained from the patient for publication of this case report and any accompanying images. A copy of the written consent is available for review by the Editor-in-Chief of this journal.

## Competing interests

The authors declare that they have no competing interests.

## Authors' contributions

Both RB and SRRY made substantive intellectual contributions to this study. Both authors analyzed and interpreted the data from our patient. RB performed all the assessments, treated our patient and was a major contributor to writing the manuscript. SRRY was involved in revising the manuscript critically for important intellectual content. Both authors have read and given approval for the final version of the manuscript to be published. Both authors participated sufficiently in the work and take public responsibility for appropriate portions of the content.
